# Review of the Function of the Hypothalamic–Pituitary–Gonadal Axis in Children and Adolescents with Cancer

**DOI:** 10.17925/EE.2022.18.2.122

**Published:** 2022-11-29

**Authors:** Jimena Lopez Dacal, Romina P Grinspon, Rodolfo A Rey

**Affiliations:** 1. Centro de Investigaciones Endocrinolègicas “Dr. César Bergadá” (CEDIE), CONICET – FEI – Divisièn de Endocrinología, Hospital de Niños Ricardo Gutiérrez, Buenos Aires, Argentina; 2. Departamento de Histología, Biología Celular, Embriología y Genética, Facultad de Medicina, Universidad de Buenos Aires, Buenos Aires, Argentina

**Keywords:** Childhood, gonadal function, malignancy, ovary, testis, hypothalamic–pituitary–gonadal axis

## Abstract

The most common malignancies in childhood are leukaemias, brain tumours, lymphomas, neuroblastomas, soft tissue sarcomas and kidney tumours. At present, about 80% of childhood cancers can be treated successfully, which has significantly increased long-term survival. Concomitantly, adult gonadal function in childhood cancer survivors has become a concern. However, the immediate effect of cancer and its management on the reproductive axis function has received less attention. We conducted a review of the effects of malignancies and their treatments on the gonadal axis during childhood and adolescence. Some results are controversial, probably because the analyses do not distinguish between the malignancy types, their treatments and/or the age at treatment. However, there is agreement that cancer can partially affect gonadal function before treatment, as revealed by low circulating levels of inhibin B and anti-Müllerian hormone. Subsequently, chemotherapy transiently impairs the somatic component of the gonads (i.e. testicular Sertoli cells and ovarian granulosa cells) with normalization after treatment ends. The impact of chemotherapy may persist through adulthood after more intensive chemotherapy regimens, radiotherapy and conditioning for haematopoietic stem cell transplantation, when there is a severe impairment of the somatic component of the gonads or of the stem germ cells.

The development of effective therapies for the management of cancer in childhood has led to outstanding improvements in survival over the past decades. With this, a growing population of childhood cancer survivors are subject to long-term sequelae impacting on health. Endocrine disorders, and especially reproductive function in adulthood, have become an important concern.^1^ There is a large body of evidence on the long-term effects on adults of chemotherapy and radiotherapy given during childhood.^2-4^ In particular, the term ‘gonadotoxic’ treatments usually refers to the harmful effects of chemo/radiotherapy on the germ cell population of the gonads (i.e. those producing the oocytes and spermatozoa in the adult). However, little attention has been driven to the immediate effects of cancer and its treatment observed during childhood, especially their impact on the somatic cell populations of the gonads, which are the most numerous cell types before pubertal development. In this narrative review, we discuss the existing evidence on the immediate effects of cancer and its treatment on the function of the gonadal axis during childhood and adolescence.

## Hypogonadism in the paediatric age group: Differences from hypogonadism in the adult

The most frequent concepts used to describe the physiology and nomenclature of the pathological conditions of the hypothalamic– pituitary–gonadal (HPG) axis derive from observations in the adult.^5^ Understanding the differences in the physiology of the HPG axis during postnatal development is essential for the interpretation of the clinical presentation of HPG disorders in children and adolescents.

### Developmental physiology of the hypothalamic– pituitary–gonadal axis

#### Female developmental physiology

In the adult woman of reproductive age, follicle-stimulating hormone (FSH) regulates the function of granulosa cells of the follicle while luteinizing hormone (LH) acts on theca cells and the corpus luteum. Androgens are synthesized by the theca in response to LH and aromatized to oestrogens in the granulosa cells in response to FSH.^6^ Additionally, follicles produce inhibin B, whereas the corpus luteum produces inhibin A, which exerts negative feedback on pituitary FSH.^7^ Inhibin B production increases progressively as follicles grow from 4 to 12 mm.^8^ Conversely, anti-Müllerian hormone (AMH) is predominantly secreted by small follicles ≤2 mm, showing a progressive decrease in follicles 4–8 mm to reach very low production in follicles ≥10 mm.^8–10.^ Serum AMH levels remain relatively stable during the menstrual cycle,^11^ reflecting the ovarian reserve^12^ and decline towards menopause.^13^

The newborn shows very low levels of gonadotropins and ovarian hormones at birth,^14^ and a subsequent increase with one or two peaks during the first year of life.^15,16^ This active period of the female HPG axis declines after 1 year of age, when serum LH and oestrogens become undetectable, and serum FSH and inhibin B are detectable but low.^16^ AMH remains stable during childhood,^17^ reflecting the pool of small follicles that do not progress to full maturation.

With pubertal onset, between 8 and 13 years of age, gonadotropins and local factors lead to the activation of terminal follicular growth.^18–20^ Menarche does not indicate full maturation of the HPG axis, and menstrual cycles may be irregular during the first years in normal adolescents.^21,22^. Ovulatory cycles, as detected by adequate progesterone production by the corpus luteum, represent only 15% in the first post-menarchal year and progressively increase to approximately 40% in the third year and 75% in the sixth year.^23^

At all ages, the interaction between germ cells and the somatic components of the ovarian follicles, i.e. granulosa and theca cells, is vital. Before puberty, the germ cell population is composed of primary oocytes arrested in the first meiotic division.^6^ The oocyte pool is fixed at birth, and new oogonia cannot be formed. The true ovarian reserve is represented by primordial follicles formed by primary oocytes and a single layer of flattened granulosa cells. During infancy and childhood, there is a non-cyclic recruitment of primordial follicles that progress to the primary follicle stage. These are formed by an arrested primary oocyte and proliferating granulosa cells in several layers; nonetheless, follicles do not progress beyond the medium (7–11 mm) antral stages.^24^ At puberty, germ cell division (meiosis) resumes, and granulosa and theca cells are also very active. Any insult to the oocyte triggering its apoptosis irremediably leads to follicular loss, whereas disorders primarily disrupting the somatic follicular cells may be reversible until oocyte survival is affected.

#### Male developmental physiology

In the adult male, LH drives Leydig cell testosterone secretion, whereas FSH regulates Sertoli cell function and inhibin B production. Androgens and inhibin B exert negative feedback on LH and FSH secretion, respectively.^25,26^ A concerted action of androgens and FSH is necessary for normal sperm output.

Similarly to females, the newborn males show very low levels of gonadotropins and testicular hormones at birth, which increase progressively during the first month of life.^14^ However, high gonadotropin and androgen levels persist for only the first 3–6 months.^15,27,28^ Curiously, although these hormone levels are in the same range as those observed in adults, the testes remain immature and sperm production does not occur, due to the lack of expression of the androgen receptor in Sertoli cells.^29^ During the rest of infancy and childhood, most of the HPG axis becomes quiescent as in the female. Gonadotropin levels decline: LH is very low or undetectable, resulting in a complete disappearance of functional Leydig cells and androgen synthesis. Sertoli cells remain immature but active, and their function can be detected through the determination of AMH and inhibin B in serum.^30^

With the onset of puberty, between 9 and 14 years of age, FSH initially boosts Sertoli cell proliferation, which provokes testicular enlargement beyond a volume of 4 mL. Within the testes, testosterone induces Sertoli cell maturation and adult spermatogenesis. Clinically, this is characterized by a decline in AMH and an increase in inhibin B, together with a progressive increase in testicular volume to 15–25 mL.^30,31^

The dynamics of germ and somatic cell populations of the testis differ from that of the ovary. As mentioned, Leydig cells are present in the first 3–6 months of life, then they are no longer seen until the onset of puberty. Immature Sertoli cells have proliferating capacity in response to FSH until intratesticular testosterone increases in puberty and provokes their maturation. The germ cell population is represented by spermatogonial stem cells, which can renew throughout life. Spermatogonia proliferate by mitosis during the active period of the HPG axis after birth but do not enter meiosis. During childhood, the rate of spermatogonial mitotic divisions decreases and it boosts again after pubertal onset. By then, spermatogonia can also enter meiosis to form spermatocytes, spermatids and spermatozoa. Defective function of Sertoli cells severely compromises germ cell survival and proliferation. Defective androgen production by Leydig cells results in lack of progression through meiosis but does not necessarily affect the survival of spermatogonia. Unlike the ovary, germ cell loss in the testis does not lead to seminiferous tubule or Leydig cell disappearance. Indeed, in the absence of germ cells, androgen production may be normal, and Sertoli cells can produce AMH normally. Nonetheless, inhibin B production is impaired as it needs both FSH and germ cell action on Sertoli cells.

### Hypogonadism in childhood and adolescence

From this discussion, it follows that the concept of hypogonadism needs to be adapted when applying it to the paediatric population. Based upon developmental physiology, hypogonadism can be defined as an impaired gonadal function, based on the patient’s age, which may result in a decreased secretion of sex steroids, AMH and/or inhibins, and/or a disrupted gamete production.^32^ Hypogonadism has different clinical presentations and health consequences depending on the age at which it is established, the gonadal cell population initially impaired and the level of the HPG axis primarily affected (*Table 1*).

An important concept in prepubertal patients is that, due to the central inactivation of the HPG axis, primary hypogonadism usually presents with normal gonadotropin levels (i.e. it is not usually hypergonadotropic as it is in adults). Indeed, girls with complete gonadal dysgenesis due to Turner syndrome have normal FSH and LH levels between ages 4 and 10 years,^33^ and a similar pattern is observed in anorchid boys ^34–36^ and in boys with primary hypogonadism associated with cryptorchidism,^37^ indicating that approximately 35–75% of the patients would go undiagnosed if gonadotropin elevation was required to diagnose primary gonadal failure in patients of prepubertal age.^38^

#### Female hypogonadism

The activity of the HPG axis is clinically inapparent in the female during foetal life and in childhood. Actually, girls with congenital primary hypogonadism (usually called primary ovarian insufficiency [POI] or failure [POF]) or with central hypogonadism are most usually diagnosed only at the age of puberty due to the absence of thelarche and menarche.^39–41^

**Table 1:** Incidence of malignant tumours in paediatric patients

	0–14 years old			15–19 years old	
Tumour type	WSR	RI	Tumour type	WSR	RI
Leukaemias	46.4	33.0%	Lymphomas	41.8	22.7%
Central NS	28.2	20.1%	Epithelial/Melanoma	39.5	21.4%
Lymphomas	15.2	10.8%	Leukaemias	28.5	15.4%
Sympathetic NS	10.4	7.4%	Germ cell/Gonadal	22.2	12.0%
Soft tissue sarcomas	8.9	6.3%	Central NS	19.9	10.8%
Renal	8.2	5.8%	Bone	14.4	7.8%
Bone	5.7	4.1%	Soft tissue sarcomas	12.9	7.0%
Germ cell/Gonadal	4.9	3.5%	Other	2.0	1.1%
Epithelial/Melanoma	4.6	3.3%	Renal	1.4	0.8%
Retinoblastoma	4.5	3.2%	Hepatic	1.2	0.7%
Hepatic	2.3	1.6%	Sympathetic NS	0.7	0.4%
Other	1.2	0.9%	Retinoblastoma	0.0	0.0%
TOTAL	140.5	100%	TOTAL	184.5	100%

*NS = nervous system; RI = relative incidence (tumour type/all tumours); WSR = age-standardized incidence rate (world standard population), cases per million children per year. Calculated from data obtained from Steliarova-Foucher et al.^53^*

As discussed earlier, except for the transient activation period of the gonadal axis in the first year after birth, serum determination of oestradiol, progesterone and gonadotropins gives little or no information. Conversely, since small ovarian follicles secrete AMH and inhibin B, these biomarkers may be used throughout childhood to assess the status of the female gonads.^42–44^ Low levels indicate a decreased follicular pool and undetectable levels denote the absence of functional ovarian tissue. Noteworthy, the definitions of POI or POF coined for the adult woman, based on the finding of serum concentrations of FSH >25 or 40 IU/L twice at least 1 month apart, low sex steroid levels and amenorrhoea for at least 4 months in a female aged less than 40 years,^7,39,45^ cannot be applied in paediatrics: occurrence of low sex steroids and no menstrual cycles reflects the physiological state in prepubertal girls and FSH remains below 25–40 IU/L during most of childhood in girls with primary hypogonadism.

#### Male hypogonadism

Unlike the observations in females, in males the function of the HPG axis during foetal and early postnatal life has a clinical impact. Because androgens are essential for testicular descent and penile growth during intrauterine development,^46^ and for persistent penile enlargement during the first 3–6 months of postnatal life,^47^ micropenis and cryptorchidism are suggestive of congenital hypogonadism in the male newborn.^48^ Conversely, hypogonadism acquired later in childhood may go clinically undiagnosed. Circulating sex steroid and gonadotropin levels are not helpful, and only low serum levels of AMH^37,49,50^ or inhibin B^51,52^ may be indicative of gonadal function impairment.

## Cancer in the paediatric age group

Epidemiology of cancer in children and adolescents Each year, approximately 400,000 children and adolescents below the age of 19 years are diagnosed with cancer worldwide.^53^ The epidemiology of cancer in children and adolescents differs significantly from that observed in adults. Indeed, in the paediatric population, the most common types of cancers include leukaemias, brain tumours, lymphomas, neuroblastoma, soft tissue sarcomas and kidney cancer, especially Wilms tumours, with differences according to age and world regions. In high-income countries, cancer is the second leading cause of death in children, after accidents. The overall annual incidence of cancer is approximately 141 per million under the age of 14 years and 185 per million in adolescents aged 15–19 years. There is a slightly higher incidence rate in boys than in girls, with an overall incidence sex ratio of 1.14. However, sex-specific incidence varies according to cancer type, and renal, gonadal and epithelial tumours are more frequent in girls.

In children below the age of 14 years, leukaemias represent approximately 33% of all cancer types (*Table 1*).^53^ Tumours of the central nervous system follow in frequency with 20%, lymphomas account for ∼11%, neuroblastomas represent 7%, soft tissue sarcomas and renal tumours ∼6% each, bone tumours 4%, and germ cell/gonadal tumours, epithelial tumours/melanomas and retinoblastomas account for 3–4% each.

In adolescents aged 15–19 years (*Table 1*), lymphomas are the most common tumour type, with approximately 23% of all cancers, followed by epithelial tumours/melanomas (21%), leukaemias (15%), germ cell/ gonadal tumours (12%), central nervous system tumours (11%), and bone and soft tissue sarcomas (between 7% and 8% each).^53^

### Oncological treatments in children and adolescents

At present, ∼80% of cancers occurring in children and adolescents can be diagnosed and successfully treated, although with differences amongst countries according to their economic capacity. A recent study comparing the global and regional burden of cancer in children 0–4 years old between 1990 and 2019 found that although the decrease in incident and prevalent cases was only 4.6% and 8.3%, respectively, the numbers of deaths and disability-adjusted life-years clearly declined by 47.8% and 47.7%, respectively.^54^ These advances in the management of childhood malignancies have significantly increased long-term survival and, concomitantly, the burden of long-term morbidity has raised concern. Approximately 40–50% of childhood cancer survivors develop an endocrine disorder in their lifetime.^55^ In particular, whether gonadal function is affected depends on risk factors including the sex and age of the patient, the diagnosis and its associated therapy.

#### Haematological malignancies

Hematological malignancies represent approximately 40% of cancers in the paediatric population,^56,57^ including acute lymphoblastic leukaemia (ALL), acute myeloid leukaemia (AML) and non-Hodgkin lymphoma (NHL). Successful treatment currently occurs in 90% of cases, based on the use of intensive multi-drug, induction and consolidation regimens.^57^

**Figure 1:**
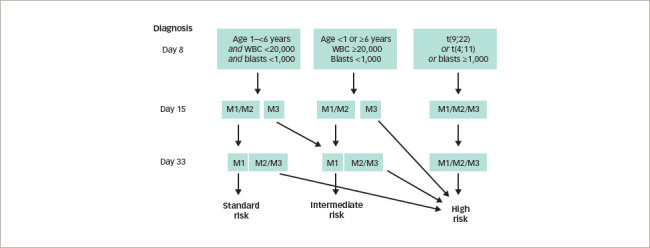
Risk stratification for patients with acute lymphoblastic leukaemia (ALL) according to the criteria set by the ALL IC-BFM 2002 trial^58,78^

*ALL IC-BFM 2002: Acute Lymphoblastic Leukemia Intercontinental Berlin-Frankfurt-Münster 2002. Standard risk: prednisone good response (<1×10^9^/L blasts in peripheral blood on day 8 after 7 days of prednisone and one dose of intrathecal methotrexate on day 1), age 1–6 years, initial WBC <20×10^9^/L, and M1 (<5% blasts) or M2 (5–25% blasts) marrow on day 15, and M1 marrow on day 33.*

*Intermediate risk: prednisone good response, age <1 year or ≥6 years, and/or WBC ≥20×10^9^/L and M1 or M2 marrow on day 15 and M1 marrow on day 33, or standard-risk criteria but M3 (≥25% blasts) marrow on day 15 and M1 marrow on day 33.*

*High risk: prednisone poor response (≥1×10^9^/L blasts in peripheral blood on day 8 after 7 days of prednisone and one dose of intrathecal methotrexate on day 1), or intermediate risk and M3 marrow on day 15, or M2 or M3 marrow on day 33, or existence of chromosomal rearrangements t(9;22) (BCR-ABL) or t(4;11) (MLL-AF4).*

*M1 = <5% bone marrow blasts; M2 = 5–25% bone marrow blasts; M3 = >25% bone marrow blasts; WBC = white blood cells.*

*Modified with permission from: Grinspon et al.^59^, copyright 2019 The Authors. Published by Oxford University Press on behalf of the European Society of Human Reproduction and Embryology.*

In the case of ALL, risk stratification and treatment are usually assigned following the criteria set by the ALL IC-BFM 2002 trial (Risk-adjusted combination chemotherapy in treating young patients with acute lymphoblastic leukemia; ClinicalTrials.gov Identifier: NCT00764907) (*Figure 1*).^58^ There are three risk groups: standard, intermediate and high risk. These are based on blast response to prednisone and intrathecal methotrexate, patient age, initial white blood cell count, percentage of blasts in bone marrow, and the presence of one of two chromosomal rearrangements: the t(9;22) translocation resulting in the fusion of the ABL proto-oncogene located on chromosome 9q34 with the breakpoint cluster region (BCR) located on 22q11.2 and giving rise to the formation of the Philadelphia chromosome (BCR-ABL); or the t(4; 11) translocation where the mixed-lineage leukaemia (*MLL*) gene on chromosome 11q23 fuses with the AF4 gene in chromosome 4q21 producing a chimeric MLL/ AF4 fusion transcript.

Standard- and intermediate-risk ALL and AML usually respond to less aggressive chemotherapy and do not require preconditioning chemotherapy for haematopoietic stem cell transplantation (HSCT) or HSCT conditioned with total body irradiation (TBI).^59^ Conversely, high-risk ALL and NHL may need HSCT with TBI or preconditioning chemotherapy including high cumulative doses of alkylating agents (e.g. busulphan, cyclophosphamide, melphalan or thiotepa).^60^ Patients with T cell ALL and high-risk patients aged 1 year or more usually receive 12 Gy prophylactic cranial radiotherapy, whereas children with initial involvement of the central nervous system receive therapeutic cranial radiotherapy with age adjustment: 12 Gy between 1 and 2 years old and 18 Gy for those older than 2 years.^58^ Gonadal irradiation is required only in cases of leukaemic gonadal relapse.

#### Solid tumours

Brain tumours are treated with surgery and may need radiation therapy. The tumour itself or the surgical procedure may affect the hypothalamic– pituitary region. The radiation dose impairs endocrine function variably: growth hormone deficiency and precocious puberty can be seen with cranial radiotherapy doses ≥18 Gy, whereas other endocrine axis manifestations require doses ≥30 Gy.^55^ Tumours of the sympathetic nervous system are treated with spinal irradiation, which may affect the hypothalamic–pituitary region or the gonads, depending on the location of the tumour.

Soft tissue sarcomas stage I, renal (Wilms) and germ cell tumours may only require surgery. When no radiotherapy is required, the risk of endocrine compromise is low. Conversely, the risk increases in the cases of metastatic or high-risk sarcomas requiring high cumulative doses of alkylating agents (cyclophosphamide, ifosfamide) and testicular irradiation.^60^

## Consequences of paediatric malignancies on hypothalamic–pituitary–gonadal axis function

The notorious advances made in the diagnosis and management of malignancies during childhood and adolescence has led to a growing population of cancer survivors who are susceptible to long-term sequelae impacting on health issues, especially endocrine disorders.^1^ Because cancer itself, as well as chemotherapy and radiotherapy, can affect gonadal function, loss of fertility and sex hormone deficiencies have become important concerns for childhood cancer survivors. We have conducted a review on the immediate effect of the occurrence of malignancies and of their treatment on gonadal function during childhood and adolescence.

Two main characteristics of the developmental biology of the HPG axis need to be considered when analysing the potential effects of childhood malignancies and their treatments on gonadal function. One is the different mitotic activity of germ and somatic cells of the gonads between childhood and puberty. The other is the difference in the activities of the germ cells and the somatic cells between the ovary and the testis. All this may explain the differential effects of the diverse chemotherapies and radiotherapies on the HPG in childhood and adolescence. According to their mechanism of action, chemotherapy agents can be classified into ‘cell-cycle-specific’ and ‘non-cell-cycle-specific’ drugs (*Table 2*). Cell-cycle-specific agents inhibit mitosis by impairing DNA synthesis, thus affecting cells with a high proliferation rate. Germ cells involved in oogenesis and spermatogenesis during pubertal development are the most susceptible cell populations to cell-cycle-specific agents.^3,60–62^ Nonetheless, granulosa cells of small ovarian follicles and testicular spermatogonia and Sertoli cells also have a moderate proliferating activity in childhood. These cell types could also be affected by cell-cycle-specific drugs. The difference in their recovery is that the oocyte pool is fixed, with no stem cell renewal after birth, while granulosa cells, spermatogonia and Sertoli cells can renew if the gonads are not completely depleted of them by treatment. Another point to consider is that oocyte depletion results in granulosa cell exhaustion in the ovary, whereas spermatogonial depletion does not overtly affect Sertoli or Leydig cell numbers in the testis. On the other hand, non-cell-cycle-specific agents provoke direct DNA and RNA damage in both proliferating and cell-cycle-arrested cells, inducing apoptosis.^60,61^ Therefore, all gonadal cell types at any age would be susceptible to these agents.

**Table 2:** Chemotherapy drugs affecting gonadal function used in boys and adolescents

Relation to cell cycle	Mechanism of action	Drug class	Drug	Risk of prolonged infertility in adulthood
Non-cell-cycle specific	Direct damage of nucleic acids (DNA and RNA) Induction of apoptosis	Alkylating agents	Cyclophosphamide Ifosfamide Busulfan Melphalan Chlorambucil Procarbazine BCNU CCNU	High
	Induction of DNA strand breaks	Antitumour antibiotics	Bleomycin Actinomycin	Low
Cell-cycle specific	Inhibition of nucleic acid synthesis (DNA and RNA) Inhibition of cell proliferation (mitosis and meiosis) Deamination of proteins	Antimetabolites	Methotrexate Mercaptopurine	Low
		Vinca alkaloids	Vincristine Vinblastine	Low
		Podophyllotoxins	Asparaginase	Low

*BCNU = bischloroethyl nitrosourea; CCNU = chloroethyl cyclohexyl nitrosourea.*

### Girls

Initially, results from studies carried out with cohorts of adult survivors of childhood cancer drove the attention to the long-term deleterious effects of chemotherapy or radiotherapy on reproductive function.^1,3,55,63^ Results of cohorts treated decades ago^64,65^ may no longer be applicable at present given the changes in the diagnostic and therapeutic alternatives available. Furthermore, more recent observations have also highlighted the effect of the health status of children with malignancies at the moment of diagnosis on the function of the HPG axis. The published results from the largest cohorts of girls with childhood cancer focused on the function of the HPG axis are summarized in *Table 3*. Four studies assessed ovarian function from the time of diagnosis (i.e. pretreatment), until several months or years after treatment end, and those still of paediatric ages.^66–69^ One cross-sectional study focused on the ovarian status at the time of diagnosis,^70^ whereas two other studies evaluated the effect of cancer and its treatment only after the end of therapy.^71,72^

Results may seem somewhat controversial regarding certain endpoints, and this could be due to the fact that some studies do not distinguish in their analyses the potential effects of the different underlying malignancies, their treatments and/or the age (prepubertal or pubertal) at which treatment was initiated and finished. However, most studies conclude that ovarian function is mildly impaired at the time of diagnosis, before any treatment is initiated.^67–70^ Brougham et al^67^ were the first to report in 22 girls (17 prepubertal, five pubertal) with solid malignancies requiring treatment with pulsed chemotherapy or radiotherapy that serum AMH was slightly but not significantly below that of the reference population mean. The analysis – which did not discriminate between tumour type or consider the duration of treatment (5–39 months) or the length of follow-up (4–43 months) - found that serum AMH was less affected in older girls, declined significantly during the initial six treatment cycles, and recovered to pre-treatment values in girls after >12 months of the end of treatment. Serum inhibin B was also below the mean at diagnosis and declined significantly in the first 4 cycles of oncological treatment. Although its circulating levels did not recover during followup, serum FSH did not increase. This could be due to the fact that most of the girls were still younger than 9 years. As already mentioned, it has been known for a long time that primary ovarian insufficiency can present with normal FSH in girls of prepubertal age.^33^

The study by van Dorp et al^70^ in 114 girls aged <18 years with haematological malignancies and 94 with solid tumours clearly showed that serum AMH is reduced at the time of diagnosis, in correlation with impairment of general health status in girls. The authors suggest that other factors, besides small follicle number, may influence ovarian AMH secretion.

In a small series of nine prepubertal girls with haematological and solid malignancies, Crofton et al.^66^ could not detect any impairment in ovarian function at diagnosis but showed a decline of serum inhibin B to undetectable levels during chemotherapy, with a variable degree of recovery 1–6 months after the end of treatment. In agreement with the study by Brougham et al.,^67^ Crofton et al did not find any significant changes in gonadotropin levels.

In a more recent and larger study, van der Kooi et al.^69^ assessed at diagnosis 129 girls aged <18 years with cancer, of whom 49 were followed during and after treatment. The analysis did not distinguish between haematological (n=31) and solid (n=18) malignancies. Serum levels of AMH were in the lowest part of the reference range in both premenarcheal and postmenarcheal girls at diagnosis. In premenarcheal girls undergoing standard-risk chemotherapy, no significant changes were seen in serum AMH during treatment and an increase was found during follow-up ≥12 months after treatment end. Conversely, serum AMH declined and did not recover to pretreatment levels in girls receiving high-risk chemotherapy. In postmenarcheal patients, serum AMH had already decreased significantly 1 month after treatment start and remained low after treatment end. The authors concluded that gonadotoxicity occurs more frequently in postmenarcheal girls and that premenarcheal girls are more likely to recover the function of the HPG axis. Therefore, they hypothesize that preventive treatment with gonadotropin hormone-releasing hormone analogues is useful to preserve ovarian function.

**Table 3:** Hypothalamic–pituitary–ovarian axis function in girls with cancer

Study reference	Inclusion criteria	Exclusion criteria	Study design, treatment and follow-up	Main results
Crofton et al. (2003)^66^	Prepubertal girls, haematological or solid malignancies	Germ cell tumours	Longitudinal, chemotherapy, lumbar radiotherapy, analysis from diagnosis to 1–6 months after end of treatment	Analysis not distinguishing between malignancy types (n=9) Inhibin B: pretreatment normal, declined to undetectable during chemotherapy, variable recovery FSH and LH: pretreatment normal, no significant changes during or after treatment
Cuny et al. (2011)^71^	Age <11 years, medulloblastoma or ependymoma	NA	Longitudinal, surgery, cranial irradiation, spinal irradiation, chemotherapy, analysis 75–166 months after end of treatment	Analysis only after pubertal age, 13–16 years (n=22) Early puberty requiring gonadotropin hormone-releasing hormone analogue treatment associated with radiotherapy (n=10) Clinical progression of puberty normal in 15 girls: normal FSH and LH in 14 and high in 1, normal AMH and inhibin B in 11, low in 4 Abnormal progression of puberty in 7 girls: 2 with central hypogonadism (low FSH and LH), 4 with primary hypogonadism (low AMH and inhibin B, high FSH and LH), and 1 with combined (central and ovarian) hypogonadism (low AMH and inhibin B with inadequately normal FSH and LH)
Brougham et al. (2012)^67^	Age <18 years, haematological or solid malignancies	ALL, brain tumours, gonadal tumours and primary gonadal dysgenesis	Longitudinal, chemotherapy, spinal or abdominal radiotherapy for 5–39 months, analysis 4–43 months after end of treatment	Analysis not distinguishing between malignancy types in prepubertal (n=17) and pubertal (n=5) AMH: pretreatment normal/low, positive correlation with age, declined during first 6 treatment cycles Inhibin B: pretreatment normal/undetectable, declined during first 4 treatment cycles FSH: pretreatment normal, no significant changes during treatment
Mörse et al. (2013)^68^	Age <18 years, haematological or solid malignancies	Brain tumours, death shortly after diagnosis, ovarian cancer, contraceptives, Down syndrome	Longitudinal, chemotherapy, abdominal radiotherapy, HSCT, analysis at diagnosis and 0–36 months after end of treatment	Analysis not distinguishing premenarche (n=23) and postmenarche (n=11), or between solid (n=23) and haematological (n=11) malignancies AMH: pretreatment normal/low, positive correlation with age, declined during first 3 months of treatment, normalized in low-dose chemotherapy, undetectable in radiotherapy and HSCT Inhibin B: pretreatment normal/undetectable, declined during first 3 months of treatment, normalized in low-dose chemotherapy, undetectable in radiotherapy and HSCT FSH, LH and E2: pre-treatment normal, increase in FSH and LH, and variable changes in E2 at 3 months. No further changes thereafter
van Dorp et al. (2014)^70^	Age <18 years, haematological or solid malignancies	Brain tumours, germ cell tumours	Cross-sectional study at diagnosis (no follow-up)	114 haematological malignancies, 94 solid tumours AMH: pretreatment lower than in normal age-matched controls, associated with impaired general health status. No relation with age or malignancy type
Wędrychowicz et al. (2017)^72^	Patients requiring HSCT for haematological or solid malignancies		Cross-sectional, HSCT (including TBI), abdominal radiotherapy, analysis 6–132 months after end of treatment	6 ALL, 4 neuroblastoma, 1 NHL, 1 HD. HSCT: 6 auto, 6 allo AMH: undetectable or extremely low in all Auto: 1 eugonadism (normal FSH, LH and inhibin B), 1 central hypogonadism (low FSH, LH and inhibin B), 2 primary hypogonadism (high FSH and LH, low/normal inhibin B), 2 combined hypogonadism (low inhibin B, inadequately normal FSH and LH)
van der Kooi et al. (2019)^69^	Age <18 years, haematological or solid malignancies	NA	Longitudinal, chemotherapy, cranial, chest, abdominal radiotherapy, analysis from diagnosis to 10 months after end of treatment	Analysis not distinguishing between haematological (n=31) and solid (n=18) malignancies AMH: pretreatment normal/low in premenarche and postmenarche. No changes during treatment and increased after treatment in premenarche standard risk; declined with no recovery in premenarche high risk. Decreased after 1 month of treatment with no recovery after treatment in postmenarche

*ALL = acute lymphoblastic leukaemia; AMH = anti-Müllerian hormone; E2 = oestradiol; FSH = follicle-stimulating hormone; HD = Hodgkin disease; HSCT = haematopoietic stem cell transplantation; LH = luteinizing hormone; NA = data not available; NHL = non-Hodgkin lymphoma; TBI = total body irradiation.*

Gonadal infiltration is one possibility to explain primary hypogonadism (ovarian dysfunction) before the start of treatment. The incidence of gonadal infiltration was high several decades ago.^73^ However, it is extremely infrequent nowadays, as revealed by an immunohistochemical assessment performed in a series of cryopreserved ovarian cortex samples obtained for fertility preservation before chemotherapy or radiotherapy from patients with leukaemia. Nevertheless, in approximately 75% of the cases, assessment of leukaemia genetic biomarkers in the ovarian tissue by polymerase chain reaction suggested the existence of leukaemic cells in the cryopreserved samples.^74^ Although their viability and malignancy is uncertain, the reimplantation of cryopreserved ovarian tissue cannot be recommended in patients with leukaemia.

High-risk treatments include those requiring HSCT. In a recent study, Wędrychowicz et al^72^ assessed ovarian function in girls who underwent HSCT. The study included six girls 6–11 years old with ALL, who received allo-HSCT, and six girls 5 months to 13 years old with neuroblastoma, Hodgkin disease or NHL, treated with auto-HSCT. In all cases, the ovarian reserve was severely impaired, as reflected by extremely low or undetectable serum AMH between 6 months and 11 years after HSCT. However, the HPG axis still showed partially or completely preserved function in six cases.

In 22 patients with medulloblastoma or ependymoma aged <11 years, treated with surgical resection, cranial and/or spinal irradiation and chemotherapy, Cuny et al.^71^ found that 10 patients developed early puberty in association with low-dose cranial radiotherapy and required treatment with a gonadotropin hormone-releasing hormone analogue. After followup between approximately 6 and 14 years, clinical progression of puberty was normal in 15 girls; FSH and LH were in the reference range in 14 of them and high in 1, indicating an incipient primary ovarian failure. Serum AMH and inhibin B were normal in 11 cases and low in 4, reflecting a decreased ovarian reserve that did not yet have an overt impact on the HPG axis function. On the other hand, abnormal progression of puberty was seen in 7 girls: 2 with central hypogonadism, 4 with primary hypogonadism and 1 with combined (central and ovarian) hypogonadism.

In summary, cancer partially affects ovarian function, probably by impairing the general health status. In certain malignancies, such as leukaemias, ovarian infiltration - even submicroscopic - may also explain a decline in AMH and inhibin B production. The underlying pathogenic mechanisms need to be unveiled. In most cases, chemotherapy during childhood impairs ovarian function transiently, as revealed by a decline in AMH and inhibin B levels, with a normalization after treatment end. This might be explained by an effect of cell-cycle-specific chemotherapy agents mainly on proliferating granulosa cells of small follicles without inducing an increased germ cell apoptosis (i.e. not affecting the ovarian reserve).^75^ In pubertal girls, the impact of chemotherapy could be longer lasting, as oocytes have reinitiated meiotic divisions. At any age, more intensive chemotherapy regimens, abdomino-pelvic radiotherapy and HSCT usually result in a severe and persistent damage of the ovarian tissue. Cranial radiotherapy affects the hypothalamic–pituitary axis: doses 18–30 Gy induce early HPG axis activation resulting in precocious puberty, whereas doses >30 Gy provoke gonadotropin deficiency, resulting in delayed/absent/arrested puberty.

### Boys

As in females, the first evidence of the deleterious effects of childhood cancer treatment on male reproductive function came from studies in cohorts of adult survivors.^1,3,55,63^ Here, also, the conclusions obtained from studies carried out decades ago^64,76–78^ may be outdated, owing to the progress made in diagnosis and treatment regimens. Results of the most recent studies of large cohorts of boys with cancer focused on the function of the HPG axis are summarized in *Table 4*. Two longitudinal studies assessed testicular function from the time of diagnosis, before any treatment, until several months or years after the end of treatment,^66,69^ whereas the other two longitudinal studies evaluated the effect of cancer therapy on testicular germ cells once therapy was initiated, during and after treatment.^79,80^ Two cross-sectional studies focused on testicular function at diagnosis.^81,82^ Finally, two studies analysed the effect of cancer and its treatment exclusively several months or years after treatment end, yet still in the paediatric age group.^59,83^

In a series of 16 prepubertal boys with haematological and solid malignancies, Crofton et al.^66^ could not detect any impairment in testicular function at diagnosis, during or after treatment, except for one case who received an intensive chemotherapy regimen. However, much larger studies yielded somewhat different results. Krawczuk-Rybak et al^81^ found that testicular volume and serum inhibin B were normal at diagnosis in 48 prepubertal boys - not distinguishing between those with haematological (n=34) and those with solid (n=14) malignancies - but decreased in 36 pubertal patients with either haematological (n=16) or solid (n=20) cancer with no impact on gonadotropins or testosterone. On the other hand, in a large crosssectional study, Wigny et al^82^ found normal inhibin B levels at diagnosis only in nine boys with nephroblastoma but a significantly lower mean inhibin B in serum in 215 boys with other solid tumours or haematological malignancies, not distinguishing between prepubertal and pubertal status. Moreover, the recent study by van der Kooi et al.^69^ found that serum inhibin B could be normal or low before treatment start in both prepubertal and pubertal boys with solid (n=19) or haematological (n=40) cancer and that it further decreased during the first month of therapy, with a partial recovery 10 months after the end of chemotherapy, which could be associated with cranial, chest and/or abdominal radiotherapy. Boys treated after pubertal onset seemed to be more affected than those treated at a prepubertal stage.

**Figure 2:**
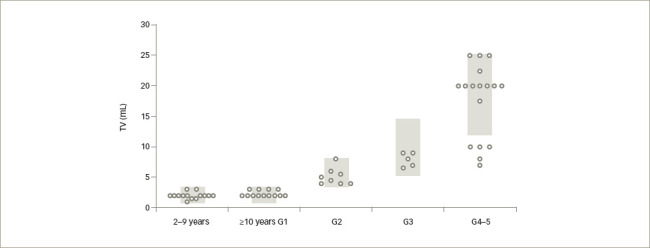
Mean bilateral testicular volume in 61 boys with acute lymphoblastic or myeloid leukaemia, or non-Hodgkin lymphoma, grouped according to age or pubertal Tanner stage at the moment of clinical assessment of testicular volume no earlier than 1 year after the end of chemotherapy

**Figure 3:**
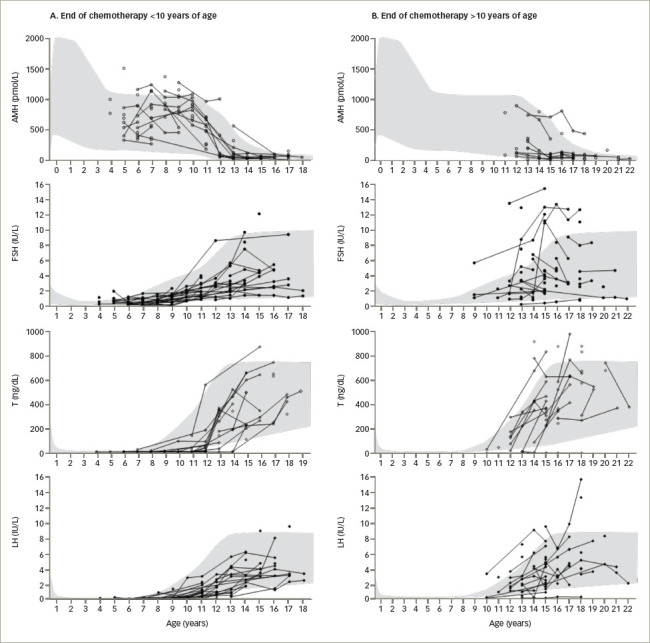
Longitudinal follow-up of serum levels of anti-Müllerian hormone, testosterone and gonadotropins in 97 boys with acute lymphoblastic or myeloid leukaemia, or non-Hodgkin lymphoma

Analysing, retrospectively, boys and adolescents who received chemotherapy - with associated cranial radiotherapy in certain cases - for leukaemia several years earlier, Krawczuk-Rybak et al.^83^ found that testicular volume was not affected in prepubertal patients but was reduced in pubertal ones; mean serum inhibin B was significantly lower independently of pubertal status, while gonadotropins and testosterone were within the normal range. Somewhat different results were reported recently by Grinspon et al.^59^ in a large series of boys with leukaemia or NHL 1–12 years after the end of chemotherapy, associated with cranial radiotherapy or HSCT, in certain cases. While testis volume was normal in boys treated before puberty, it could be affected in those treated during puberty (*Figure 2*). These results suggest that the chemotherapy regimens used, including drugs of low risk for spermatogenic toxicity and doses of alkylating agents that are below the expected toxicity risk, do not significantly affect the Sertoli cell population, which accounts for the major proportion of testicular volume in childhood. Serum AMH and testosterone were within the normal range in all cases, while gonadotropins were normal in those treated before puberty and normal or slightly elevated in those who received treatment until after pubertal onset, especially in those who received high-risk therapy (*Figure 3*). Altogether, these observations indicate that the reduced testicular volume observed during puberty is due to the impairment of the germ cell population with no major damage of the somatic cell populations of the testis.

**Table 4:** Hypothalamic–pituitary-testicular axis function in boys with cancer

Study reference	Inclusion criteria	Exclusion criteria	Study design, treatment and follow-up	Main results
Crofton et al. (2003)^66^	Prepubertal boys, haematological and solid malignancies	Germ cell tumours	Longitudinal, chemotherapy, lumbar radiotherapy, analysis from diagnosis to 1–6 months after end of treatment	Analysis not distinguishing between malignancy types (n=16) Inhibin B: pretreatment normal, no changes during chemotherapy, except for 1 case with intensive chemotherapy FSH and LH: pretreatment normal, no significant changes during or after treatment
Krawczuk-Rybak et al. (2009)^83^	Boys and adolescents, ALL	NA	Cross-sectional, chemotherapy, brain radiotherapy, analysis 1.9 ± 1.3 years (prepubertal) or 5.3 ± 3.5 years (pubertal) after end of treatment	27 prepubertal / 32 pubertal Testis volume: normal in prepubertal, reduced in pubertal Inhibin B: decreased in prepubertal and pubertal Testosterone, FSH and LH: normal in prepubertal and pubertal
Nurmio et al. (2009)^79^	Prepubertal boys, ALL	NA	Longitudinal, chemotherapy, analyses at end of induction therapy, end of chemotherapy and end of puberty	Spermatogonia: number decreased 50% in standard-risk treatment (n=12) and 80% in high-risk treatment including cyclophosphamide (n=9). Without cyclophosphamide, no significant effect on spermatogonia. Leukaemia therapy, even without cyclophosphamide, delayed the initiation of spermatogenesis Testicular volume, FSH, LH, testosterone and inhibin B at end of puberty: normal in standard-risk treatment. Variable compromise in high risk
Krawczuk-Rybak et al. (2012)^81^	Boys and adolescents, haematological or solid malignancies	CNS or gonadal involvement	Cross-sectional study at diagnosis (no follow-up)	Prepubertal (n=48) not distinguished between haematological (n=34) and solid (n=14). Pubertal (n=36), not distinguished between haematological (n=16) and solid (n=20) Testis volume: pretreatment normal in prepubertal and early pubertal; decreased in mid-late pubertal Inhibin B: pretreatment normal in prepubertal and early pubertal; decreased in mid-late pubertal Testosterone, FSH and LH: pretreatment normal in prepubertal and pubertal
Wigny et al. (2016)^82^	<18 years, haematological or solid malignancies	Brain tumours, germ cell tumours	Cross-sectional study at diagnosis (no follow-up)	Haematological (n=151), solid (n=73). Prepubertal (n=162), pubertal (n=82) Inhibin B: pretreatment normal in nephroblastoma, normal/ low in all other solid and haematological malignancies Testosterone, FSH and LH: pretreatment normal in prepubertal and pubertal
Poganitsch-Korhonen et al. (2017)^80^	Prepubertal boys, ALL undergoing testicular biopsy to examine possible testicular leukaemia at end of therapy	NA	Longitudinal, chemotherapy, CNS or testis radiotherapy, analyses at end of treatment and end of puberty	Spermatogonia: number decreased in treatment with alkylating agents compared with non-alkylating. Total depletion of spermatogonia rare, more frequent in treatment with alkylating agents End of puberty: no significant difference in testis volume, sperm count and incidence of azoospermia between alkylating and non-alkylating treatments
Grinspon et al. (2019)^59^	Age 1–18 years, haematological malignancies	CNS or gonadal involvement	Longitudinal, chemotherapy, cranial radiotherapy, HSCT, analysis 1–12 years after end of treatment	Prepubertal, standard or intermediate risk (n=47) or high risk (n=4); pubertal, standard or intermediate risk (n=28) or high risk (n=4) Testis volume: normal in prepubertal, normal or decreased in pubertal AMH and testosterone: normal in all FSH and LH: normal in prepubertal, normal or slightly elevated in pubertal
van der Kooi et al. (2019)^69^	Age <18 years, solid or haematological malignancy	NA	Longitudinal, chemotherapy, cranial, chest, abdominal radiotherapy, analysis 10 months after end of treatment	Analysis not distinguishing between haematological (n=40) and solid (n=19) malignancies Inhibin B: pretreatment normal/low in prepubertal and pubertal. Decreased after 1 month of treatment with partial recovery after treatment. Pubertal boys more affected than prepubertal

*ALL = acute lymphoblastic leukaemia; CNS = central nervous system; HSCT = haematopoietic stem cell transplantation; NA = data not available; NHL = non-Hodgkin lymphoma.*

*The grey areas represent normal reference values, according to Grinspon et al.^49^*

*Modified with permission from: Grinspon et al.^59^, copyright 2019 The Authors. Published by Oxford University Press on behalf of the European Society of Human Reproduction and Embryology.*

*TV = testicular volume*

The two studies that focused on the germ cell population confirmed the already known toxicity of alkylating agents. Nurmio et al.^79^ conducted a longitudinal study in boys with ALL. Compared with the end of induction therapy, the number of spermatogonia significantly decreased by the end of chemotherapy in boys who had received high-risk treatment including cyclophosphamide. Without cyclophosphamide, no significant effect on the number of spermatogonia was observed, but the initiation of spermatogenesis was delayed. In a more recent survey, including prepubertal boys with ALL treated with chemotherapy and cranial or testicular radiotherapy, Poganitsch-Korhonen et al.^80^ found that while the mean number of spermatogonia decreased in boys treated with alkylating agents compared with non-alkylating drugs, a complete depletion of spermatogonia is rare, thus allowing for a recovery of spermatogenesis during puberty in most cases.

In summary, Sertoli cell endocrine function of the testis may be affected by cancer during childhood and adolescence at the time of diagnosis, similarly to what is observed in ovarian endocrine function in girls. In boys, this is reflected by inhibin B levels in the low/normal range or clearly below reference levels. Chemotherapy acutely impairs Sertoli cell function, but it does not seem to affect Sertoli cell numbers, as can be deduced by the recovery of inhibin B or AMH levels in the follow-up at least 1 year after the end of treatment. Leydig cell function does not seem to be affected, as revealed by normal testosterone production during puberty. However, the slight increase in gonadotropins observed in some cases may be the reflection of a mild compromise of Sertoli and Leydig cell function. Non-cellcycle-specific drugs, such as alkylating agents, transiently affect spermatogonial stem cell numbers, although they rarely produce a complete depletion. Consequently, azoospermia is not a frequent consequence of standard therapy for haematological malignancies. Intensive (high-risk) treatments including conditioning for HSCT and testicular radiotherapy are more deleterious, and pubertal status at the time of treatment is associated with a more severe effect on the HPG axis.

## Conclusions

Compared with the extensive experience of the long-term effects of treatment on the reproductive axis in adult childhood cancer survivors, there are limited studies that have addressed the immediate effects of cancer itself and its treatment on gonadal function during childhood. In some cases, contradictory results may arise from studies not analysing separately the effects of malignancy types, treatments using drugs with different mechanisms of actions, or the pubertal status when treatment was administered. There is consensus that gonadal function may be partially affected at the time of diagnosis, before treatment is initiated, and that standard-risk chemotherapy has a transient deleterious effect on the somatic component of the gonads (i.e. Sertoli cells of the testis and granulosa cells of the ovary). The germ cell population is rarely completely depleted, which explains why gametogenesis is frequently present in young adult survivors. This raises the question of whether the concept of ‘gonadotoxic’ used for cancer treatments in adulthood should be applied identically in childhood.

Unfortunately, no established protocols exist for the assessment of the gonadal axis during the follow-up of children with cancer. Based on the concepts of the pathophysiology of hypogonadism in paediatric age groups discussed in this review and of the scarce published findings on the effects of cancer and its treatment on the gonadal axis during childhood, the clinical assessment that may be suggested for paediatric patients certainly differs from the well-established protocols used in adults. In boys, direct assessment of the germ cell population is not feasible, and the evaluation of the LH–Leydig cell axis would require stimulation tests that may not be informative. Therefore, measurement of direct serum markers of Sertoli cell function (i.e. inhibin B and AMH) seem to be the most sensible decision. Measurement of serum FSH, as an indirect marker of Sertoli cell dysfunction, may not be informative before pubertal onset. Similar concepts are applicable to the followup of prepubertal girls: serum AMH and inhibin B seem to be the most reliable markers of the function of the hypothalamic–pituitary–ovarian axis, while FSH, LH and sex steroids give little information. For pubertal girls, the reestablishment of menstrual cycles with normal levels of gonadotropins and sex steroids indicates the recovery of a functional axis, but does not necessarily predict a normal status of the ovarian follicle reserve, which may be estimated by measuring serum AMH. In summary, further research is warranted to clarify the extent and duration of the specific effect of the different chemotherapy regimens on each gonadal compartment.

*A: Patients treated before pubertal onset. B: Patients in whom chemotherapy ended after the initiation of puberty. The grey areas represent normal reference values, according to Grinspon et al.^49^*

*AMH = anti-Müllerian hormone; FSH = follicle-stimulating hormone; LH = luteinizing hormone; T = testosterone.*

*Modified with permission from: Grinspon et al.^59^, copyright 2019 The Authors. Published by Oxford University Press on behalf of the European Society of Human Reproduction and Embryology.*

## References

[1] Crowne E, Gleeson H, Benghiat H (2015). Effect of cancer treatment on hypothalamic-pituitary function.. Lancet Diabetes Endocrinol.

[2] Jahnukainen K, Stukenborg JB (2012). Clinical review: Present and future prospects of male fertility preservation for children and adolescents.. J Clin Endocrinol Metab..

[3] Gebauer J, Higham C, Langer T (2019). Long-term endocrine and metabolic consequences of cancer treatment: A systematic review.. Endocr Rev..

[4] Kesari KK, Agarwal A, Henkel R (2018). Radiations and male fertility.. Reprod Biol Endocrinol..

[5] Rey RA, Tena-Sempere M. (2020). Puberty: Recent advancements in its physiology and the management of its disorders.. Curr Opin Endocr Metab Res..

[6] Conti M, Chang RJ (2016). Folliculogenesis, Ovulation, and Luteogenesis. In: Jameson JL, De Groot LC, de Kretser D, Giudice LC, Grossman A, Melmed S, Potts JT, Jr., and Weir GC (eds).. Endocrinology: Adult and Pediatric 7th Ed. Philadelphia: Elsevier Inc.,.

[7] De Vos M, Devroey P (2010). Fauser BCJM.. Primary ovarian insufficiency. Lancet..

[8] Andersen CY, Schmidt KT, Kristensen SG (2010). Concentrations of AMH and inhibin-B in relation to follicular diameter in normal human small antral follicles.. Hum Reprod..

[9] Long WQ, Ranchin V, Pautier P (2000). Detection of minimal levels of serum anti-Müllerian hormone during follow-up of patients with ovarian granulosa cell tumor by means of a highly sensitive enzyme-linked immunosorbent assay.. J Clin Endocrinol Metab..

[10] Rey R, Sabourin JC, Venara M (2000). Anti-Mullerian hormone is a specific marker of Sertoli- and granulosa-cell origin in gonadal tumors.. Hum Pathol..

[11] Hagen CP, Aksglaede L, Sørensen K (2010). Serum levels of anti-Müllerian hormone as a marker of ovarian function in 926 healthy females from birth to adulthood and in 172 Turner syndrome patients.. J Clin Endocrinol Metab..

[12] di Clemente N, Racine C, Pierre A, Taieb J (2021). Anti-Müllerian hormone in female reproduction.. Endocr Rev..

[13] de Kat AC, Broekmans FJM, Lambalk CB (2021). Role of AMH in prediction of menopause.. Front Endocrinol (Lausanne)..

[14] Bergadá I, Milani C, Bedecarrás P (2006). Time course of the serum gonadotropin surge, inhibins, and anti-Müllerian hormone in normal newborn males during the first month of life.. J Clin Endocrinol Metab..

[15] Kuiri-Hänninen T, Dunkel L, Sankilampi U (2018). Sexual dimorphism in postnatal gonadotrophin levels in infancy reflects diverse maturation of the ovarian and testicular hormone synthesis.. Clin Endocrinol (Oxf)..

[16] Ljubicic ML, Busch AS, Upners EN (2022). A biphasic pattern of reproductive hormones in healthy female infants – The COPENHAGEN Minipuberty Study.. J Clin Endocrinol Metab..

[17] Yates AP, Jopling HM, Burgoyne NJ (2019). Paediatric reference intervals for plasma anti-Müllerian hormone: Comparison of data from the Roche Elecsys assay and the Beckman Coulter Access assay using the same cohort of samples.. Ann Clin Biochem..

[18] Gougeon A (1996). Regulation of ovarian follicular development in primates: Facts and hypotheses.. Endocr Rev..

[19] Hsueh AJW, Kawamura K, Cheng Y (2015). Fauser BCJM. Intraovarian control of early folliculogenesis.. Endocr Rev..

[20] Richards JS, Ascoli M. (2018). Endocrine paracrine, and autocrine signaling pathways that regulate ovulation.. Trends Endocrinol Metab..

[21] Rosenfield RL (2013). Clinical review: Adolescent anovulation: Maturational mechanisms and implications.. J Clin Endocrinol Metab..

[22] Sun BZ, Kangarloo T, Adams JM (2019). Healthy post-menarchal adolescent girls demonstrate multi-level reproductive axis immaturity.. J Clin Endocrinol Metab..

[23] Apter D (1998). Endocrine and metabolic abnormalities in adolescents with a PCOS-like condition: Consequences for adult reproduction.. Trends Endocrinol Metab..

[24] Kuiri-Hänninen T, Kallio S, Seuri R (2011). Postnatal developmental changes in the pituitary-ovarian axis in preterm and term infant girls.. J Clin Endocrinol Metab..

[25] Amory JK, Bremner W (2001). Endocrine regulation of testicular function in men: Implications for contraceptive development.. Mol Cell Endocrinol..

[26] Anderson RA (2001). Clinical studies: Inhibin in the adult male.. Mol Cell Endocrinol..

[27] Andersson AM, Toppari J, Haavisto AM (1998). Longitudinal reproductive hormone profiles in infants: Peak of inhibin B levels in infant boys exceeds levels in adult men.. J Clin Endocrinol Metab..

[28] Busch AS, Ljubicic ML, Upners EN (2022). Dynamic changes of reproductive hormones in male minipuberty: Temporal dissociation of Leydig- and Sertoli-cell activity.. J Clin Endocrinol Metab..

[29] Chemes HE, Rey RA, Nistal M (2008). Physiological androgen insensitivity of the fetal, neonatal, and early infantile testis is explained by the ontogeny of the androgen receptor expression in Sertoli cells.. J Clin Endocrinol Metab..

[30] Grinspon RP, Urrutia M, Rey RA (2018). Male central hypogonadism in paediatrics – The relevance of follicle-stimulating hormone and sertoli cell markers.. Eur Endocrinol..

[31] Rey RA (2014). Mini-puberty and true puberty: Differences in testicular function.. Ann Endocrinol (Paris)..

[32] Rey RA, Grinspon RP, Gottlieb S (2013). Male hypogonadism: An extended classification based on a developmental, endocrine physiology-based approach.. Andrology..

[33] Conte FA, Grumbach MM, Kaplan SL (1975). A diphasic pattern of gonadotropin secretion in patients with the syndrome of gonadal dysgenesis.. J Clin Endocrinol Metab..

[34] Lustig RH, Conte FA, Kogan BA, Grumbach MM (1987). Ontogeny of gonadotropin secretion in congenital anorchism: Sexual dimorphism versus syndrome of gonadal dysgenesis and diagnostic considerations.. J Urol..

[35] Brauner R, Neve M, Allali S (2011). Clinical, biological and genetic analysis of anorchia in 26 boys.. PLoS One..

[36] Grinspon RP, Ropelato MG, Bedecarrás P (2012). Gonadotrophin secretion pattern in anorchid boys from birth to pubertal age: Pathophysiological aspects and diagnostic usefulness.. Clin Endocrinol (Oxf)..

[37] Grinspon RP, Gosttlieb S, Bedecarras P, Rey RA (2018). Anti-Müllerian hormone and testicular function in prepubertal boys with cryptorchidism.. Front Endocrinol (Lausanne)..

[38] Grinspon RP, Freire AV, Rey RA (2019). Hypogonadism in pediatric health: Adult medicine concepts fail.. Trends Endocrinol Metab..

[39] Huhtaniemi I, Hovatta O, La Marca A (2018). Advances in the molecular pathophysiology, genetics, and treatment of primary ovarian insufficiency.. Trends Endocrinol Metab..

[40] Ladjouze A, Donaldson M (2019). Primary gonadal failure.. Best Pract Res Clin Endocrinol Metab..

[41] França MM, Mendonca BB (2022). Genetics of ovarian insufficiency and defects of folliculogenesis.. Best Pract Res Clin Endocrinol Metab..

[42] Gravholt CH, Naeraa RW, Andersson AM (2002). Inhibin A and B in adolescents and young adults with Turner’s syndrome and no sign of spontaneous puberty.. Hum Reprod..

[43] Hagen CP, Mouritsen A, Mieritz MG (2015). Circulating AMH reflects ovarian morphology by magnetic resonance imaging and 3D ultrasound in 121 healthy girls.. J Clin Endocrinol Metab..

[44] Messina MF, Aversa T, Salzano G (2015). Inhibin B in adolescents and young adults with Turner syndrome.. J Pediatr Endocrinol Metab..

[45] Pastore LM, Christianson MS, Stelling J (2018). Reproductive ovarian testing and the alphabet soup of diagnoses: DOR, POI, POF, POR, and FOR.. J Assist Reprod Genet..

[46] Klonisch T, Fowler PA, Hombach-Klonisch S (2004). Molecular and genetic regulation of testis descent and external genitalia development.. Dev Biol..

[47] Boas M, Boisen KA, Virtanen HE (2006). Postnatal penile length and growth rate correlate to serum testosterone levels: A longitudinal study of 1962 normal boys.. Eur J Endocrinol..

[48] Braslavsky D, Grinspon RP, Ballerini MG (2015). Hypogonadotropic hypogonadism in infants with congenital hypopituitarism: A challenge to diagnose at an early stage.. Horm Res Paediatr..

[49] Grinspon RP, Bedecarrás P, Ballerini MG (2011). Early onset of primary hypogonadism revealed by serum anti-Müllerian hormone determination during infancy and childhood in trisomy 21.. Int J Androl..

[50] Weintraub A, Eldar-Geva T (2017). Anti-Müllerian hormone (AMH) determinations in the pediatric and adolescent endocrine practice.. Pediatr Endocrinol Rev..

[51] Andersson AM (2000). Inhibin B in the assessment of seminiferous tubular function.. Baillieres Best Pract Res Clin Endocrinol Metab..

[52] Condorelli RA, Cannarella R, Calogero AE, La Vignera S (2018). Evaluation of testicular function in prepubertal children.. Endocrine..

[53] Steliarova-Foucher E, Colombet M, Ries LAG (2017). International incidence of childhood cancer, 2001–10: A population-based registry study.. Lancet Oncol..

[54] Ren HM, Liao MQ, Tan SX (2022). Global, regional, and national burden of cancer in children younger than 5 years, 1990-2019: Analysis of the Global Burden of Disease Study 2019.. Front Public Health..

[55] Sklar CA, Antal Z, Chemaitilly W (2018). Hypothalamic-pituitary and growth disorders in survivors of childhood cancer: An Endocrine Society Clinical Practice Guideline.. J Clin Endocrinol Metab..

[56] Armstrong GT, Chen Y, Yasui Y (2016). Reduction in late mortality among 5-year survivors of childhood cancer.. N Engl J Med..

[57] Hunger SP, Mullighan CG (2015). Acute lymphoblastic leukemia in children.. N Engl J Med..

[58] Stary J, Zimmermann M, Campbell M (2014). Intensive chemotherapy for childhood acute lymphoblastic leukemia: Results of the randomized intercontinental trial ALL IC-BFM 2002.. J Clin Oncol..

[59] Grinspon RP, Arozarena M, Prada S (2019). Safety of standardised treatments for haematologic malignancies as regards to testicular endocrine function in children and teenagers.. Hum Reprod..

[60] Jahnukainen K, Ehmcke J, Hou M, Schlatt S (2011). Testicular function and fertility preservation in male cancer patients.. Best Pract Res Clin Endocrinol Metab..

[61] Stukenborg JB, Jahnukainen K, Hutka M, Mitchell RT (2018). Cancer treatment in childhood and testicular function: The importance of the somatic environment.. Endocr Connect..

[62] Mauri D, Gazouli I, Zarkavelis G (2020). Chemotherapy associated ovarian failure.. Front Endocrinol (Lausanne)..

[63] van Iersel L, Mulder RL, Denzer C (2022). Hypothalamic-pituitary and other endocrine surveillance among childhood cancer survivors.. Endocr Rev..

[64] Pasqualini T, Diez B, Domené H (1987). Long-term endocrine sequelae after surgery, radiotherapy, and chemotherapy in children with medulloblastoma.. Cancer..

[65] Pasqualini T, Escobar ME, Domené H (1987). Evaluation of gonadal function following long-term treatment for acute lymphoblastic leukemia in girls.. Am J Pediatr Hematol Oncol..

[66] Crofton PM, Thomson AB, Evans AE (2003). Is inhibin B a potential marker of gonadotoxicity in prepubertal children treated for cancer?. Clin Endocrinol (Oxf)..

[67] Brougham MF, Crofton PM, Johnson EJ (2012). Anti-Müllerian hormone is a marker of gonadotoxicity in pre- and postpubertal girls treated for cancer: A prospective study.. J Clin Endocrinol Metab..

[68] Mörse H, Elfving M, Lindgren A (2013). Acute onset of ovarian dysfunction in young females after start of cancer treatment.. Pediatr Blood Cancer..

[69] van der Kooi ALLF, van den Heuvel-Eibrink MM, van den Berg SAA (2019). Changes in anti-Müllerian hormone and inhibin B in children treated for cancer.. J Adolesc Young Adult Oncol..

[70] van Dorp W, van den Heuvel-Eibrink MM, de Vries AC (2014). Decreased serum anti-Müllerian hormone levels in girls with newly diagnosed cancer.. Hum Reprod..

[71] Cuny A, Trivin C, Brailly-Tabard S (2011). Inhibin B and anti-Müllerian hormone as markers of gonadal function after treatment for medulloblastoma or posterior fossa ependymoma during childhood.. J Pediatr..

[72] Wędrychowicz A, Wojtyś J, Starzyk J (2017). Anti-Muellerian hormone (AMH) as only possible marker in the assessment of ovarian function and reserve after hematopoietic stem cell transplantation (HSCT) in prepubertal girls, young females with composed hypogonadism and females receiving hormonal replacement therapy.. Bone Marrow Transplant..

[73] Reid H, Marsden HB (1980). Gonadal infiltration in children with leukaemia and lymphoma.. J Clin Pathol..

[74] Rosendahl M, Andersen MT, Ralfkiær E (2010). Evidence of residual disease in cryopreserved ovarian cortex from female patients with leukemia.. Fertil Steril..

[75] Feng J, Ma W-W, Li H-X (2022). Melatonin prevents cyclophosphamide-induced primordial follicle loss by inhibiting ovarian granulosa cell apoptosis and maintaining AMH expression.. Front Endocrinol (Lausanne)..

[76] Lendon M, Hann IM, Palmer MK (1978). Testicular histology after combination chemotherapy in childhood for acute lymphoblastic leukaemia.. Lancet..

[77] Shalet SM, Hann IM, Lendon M (1981). Testicular function after combination chemotherapy in childhood for acute lymphoblastic leukaemia.. Arch Dis Child..

[78] Pasqualini T, Chemes H, Domené H (1983). Evaluation of testicular function following long-term treatment for acute lymphoblastic leukemia.. Am J Pediatr Hematol Oncol..

[79] Nurmio M, Keros V, Lähteenmäki P (2009). Effect of childhood acute lymphoblastic leukemia therapy on spermatogonia populations and future fertility.. J Clin Endocrinol Metab..

[80] Poganitsch-Korhonen M, Masliukaite I, Nurmio M (2017). Decreased spermatogonial quantity in prepubertal boys with leukaemia treated with alkylating agents.. Leukemia..

[81] Krawczuk-Rybak M, Płonowski M, Solarz E (2012). Assessment of gonadal function in boys and adolescents at the diagnosis of neoplastic disease.. J Pediatr Endocrinol Metab..

[82] Wigny KMGJ, van Dorp W, van der Kooi ALLF (2016). Gonadal function in boys with newly diagnosed cancer before the start of treatment.. Hum Reprod..

[83] Krawczuk-Rybak M, Solarz E, Wysocka J (2009). Testicular function after treatment for acute lymphoblastic leukemia (ALL) in prepubertal and pubertal boys.. Pediatr Hematol Oncol..

